# Sugar metabolism and accumulation in the fruit of transgenic apple trees with decreased sorbitol synthesis

**DOI:** 10.1038/s41438-018-0064-8

**Published:** 2018-12-01

**Authors:** Mingjun Li, Pengmin Li, Fengwang Ma, Abhaya M. Dandekar, Lailiang Cheng

**Affiliations:** 1 State Key Laboratory of Crop Stress Biology in Arid Areas/Shaanxi Key Laboratory of Apple, College of Horticulture, Northwest A&F University, 712100 Yangling, Shaanxi P. R. China; 2 Section of Horticulture, School of Integrative Plant Science, Cornell University, Ithaca, NY 14853 USA; 3 Department of Plant Sciences, University of California, Davis, CA 95616 USA

## Abstract

Both sorbitol and sucrose are synthesized in source leaves and transported to fruit for supporting fruit growth in tree fruit species of the Rosaceae family. In apple (*Malus domestica*), antisense suppression of *aldose-6-phosphate reductase*, the key enzyme for sorbitol synthesis, significantly decreased the sorbitol concentration but increased the sucrose concentration in leaves, leading to a lower sorbitol but a higher sucrose supply to fruit in these plants. In response to this altered carbon supply, the transgenic fruit had lower concentration of sorbitol and much higher concentration of glucose but similar levels of fructose, sucrose, and starch throughout fruit development relative to the untransformed control. Activities of sorbitol dehydrogenase, fructokinase, and sucrose phosphate synthase were lower, whereas activities of neutral invertase, sucrose synthase, and hexokinase were higher in the transgenic fruit during fruit development. Transcript levels of *MdSOT1*, *MdSDHs*, *MdFK2*, and *MdSPS3/6* were downregulated, whereas transcript levels of *MdSUC1/4*, *MdSUSY1-3*, *MdNIV1/3*, *MdHK*s, and *MdTMT1* were upregulated in the transgenic fruit. These findings suggest that the Sucrose cycle and the sugar transport system are very effective in maintaining the level of fructose and provide insights into the roles of sorbitol and sucrose in regulating sugar metabolism and accumulation in sorbitol-synthesizing species.

## Apple fruit: The advantages of a flexible sugar cycle

Analysis of the sugar metabolism and transport system in apple trees shows how the fruit fructose level and fruit production is largely maintained in response to decreased sorbitol synthesis in leaves. Disruption to developmental processes and environmental factors can alter the supply of sugars from leaves to fruit. Lailiang Cheng at Cornell University, New York, US, and co-workers examined sugar metabolism and transport in modified apple trees to ascertain how a deficiency in one sugar, sorbitol, influences fruit development and sugar accumulation. Ordinarily, fructose—the sugar that generates sweeter fruit—is mainly derived from sorbitol. The modified trees compensated for sorbitol loss by increasing their sucrose supply and converting the excess sucrose into fructose and glucose, largely sustaining fruit production and fructose level. The team’s results demonstrate the metabolic flexibility of the sugar cycle in fleshy fruits.

## Introduction

In fleshy fruits, soluble sugars, including sucrose, fructose, and glucose, are not only essential for fruit growth and development but also central to fruit quality. Fruit taste and flavor is closely related to the composition and concentration of sugars and their balance with acids^1,2^. As the composition and concentration of sugars at fruit maturity is determined by metabolic and transport processes during fruit development, understanding these processes and their regulation is important for fruit quality improvement.

At the center of sugar metabolism in sink cells is the Sucrose cycle, previously named the Sucrose–Sucrose cycle^3^ or the futile Sucrose recycle^4^, which consists of the breakdown of sucrose by invertase and sucrose synthase, the phosphorylation of the resulting hexoses and the interconversion between hexose phosphates and UDP-glucose, and the re-synthesis of sucrose via sucrose-6-phosphate synthase (SPS) and sucrose-6-phosphate phosphatase. This metabolic system connects sugar metabolism with many other metabolic pathways such as glycolysis and tricarboxylic acid cycle, starch synthesis, and cellulose synthesis, and its coordination with the sugar transport system on the tonoplast is expected to determine the partitioning of sugars between metabolism in the cytosol and accumulation in the vacuole^3^. In fleshy fruits, the concentration and distribution of sugars in parenchyma cells are affected via this cycle by developmental processes^3,5–8^ and environmental factors^9^. However, the biochemical regulation of the cycle and the associated transport system is not fully understood.

Apple (*Malus domestica* Borkh.) is one of the most economically important deciduous tree fruits worldwide. In apple and many other tree fruit species of the Rosaceae family, sorbitol is a primary end product of photosynthesis and a major phloem-translocated carbohydrate, accounting for 60–80% of the photosynthates produced in apple leaves and transported in the phloem^10–13^. In source leaves, sorbitol is synthesized from glucose-6-phosphate (G6P) in a two-step process: G6P is first converted to sorbitol-6-phosphate (S6P) via aldose-6-phosphate reductase (A6PR), then followed by dephosphorylation of S6P to sorbitol via S6P phosphatase^14,15^. The loading of both sorbitol and sucrose into the companion cell-sieve element (SE-CC) complex in the phloem is passive and symplastic in apple^16,17^, but their phloem unloading in fruit involves an apoplastic step^18^. Once released from the SE-CC complex of the phloem in apple fruit, sorbitol is taken up into the cytosol of parenchyma cells by plasma membrane-bound sorbitol transporters (SOTs) and then converted to fructose by sorbitol dehydrogenase (SDH, EC 1.1.1.14); sucrose is either directly taken up into parenchyma cells by sucrose transporters (SUC; SUT), or first converted to glucose and fructose by cell wall invertase (CWINV) and then transported into the parenchyma cells via hexose transporters^18^. Compared with plants that transport and utilize only sucrose, such as *Arabidopsis*, tomato (*Solanum lycopersicon*), and poplar (*Populus*), apple is unique in that both sorbitol and sucrose are transported in the phloem and are metabolized in sink organs. It is estimated that >80% of the total carbon flux goes through fructose in apple^3^. Once taken up into parenchyma cells of fruit, both sorbitol and sucrose feed into the Sucrose cycle to meet the carbon requirement for fruit growth and development while excess carbon is converted to starch for storage in plastids or transported into vacuole by sugar transporters for accumulation. Although we have characterized the genes and proteins involved in sugar metabolism and accumulation in apple^3,8,19^, it remains unclear how apple trees adjust the Sucrose cycle and the transport system in response to altered supply of sorbitol and sucrose from source leaves.

In transgenic apple trees with antisense suppression of *A6PR*, leaf sorbitol concentration is dramatically decreased, whereas sucrose concentration is significantly elevated in the source leaves, but neither leaf CO_2_ assimilation nor plant vegetative growth is altered^13^. The decreased sorbitol synthesis leads to significant changes in the expression profile of key genes in leaf starch metabolism and many stress response genes^20^. In addition to being a key metabolite in carbohydrate metabolism, sorbitol also acts as a signal regulating stamen development and pollen tube growth and resistance to *Alternaria alternata* in apple^21,22^. In the shoot tips of the *A6PR* transgenic plants, both the activity and transcript level of SDH are downregulated, whereas those of sucrose synthase (SUSY) are upregulated in response to a lower sorbitol but higher sucrose supply^23^. Teo et al.^24^ reported that fruit of the transgenic apple trees accumulated a higher level of glucose and lower levels of fructose and starch at maturity, but no significant difference was detected in the activity of key enzymes in sugar metabolism, CWINV, neutral invertase (NINV), fructokinase (FK), hexokinase (HK), or SPS between the transgenic lines and the untransformed control (CK). Considering that (1) antisense suppression of *A6PR* has drastically decreased leaf sorbitol level and increased sucrose level^13,23,24^, leading to less sorbitol but more sucrose being transported in the phloem^24^; and (2) both transcript levels and activities of SDH and SUSY responded to the altered sorbitol and sucrose supply in the shoot tips of the transgenic plants^23^, we predicted that the decreased supply of sorbitol and increased supply of sucrose would lead to downregulation of sorbitol metabolism and upregulation of sucrose metabolism in the transgenic fruit as well. The discrepancy between the data obtained by Teo at al.^24^ and our predicted responses on the activities of sucrose-metabolizing enzymes in the transgenic fruit has prompted us to re-evaluate sugar metabolism and accumulation in the fruit of these transgenic plants to better understand how the Sucrose cycle and the sugar transport system respond to an altered supply of sorbitol and sucrose.

## Results

### Sugar concentrations in the source leaves, leaf petioles, and fruit pedicels of the transgenic apple trees

**Fig. 1:**
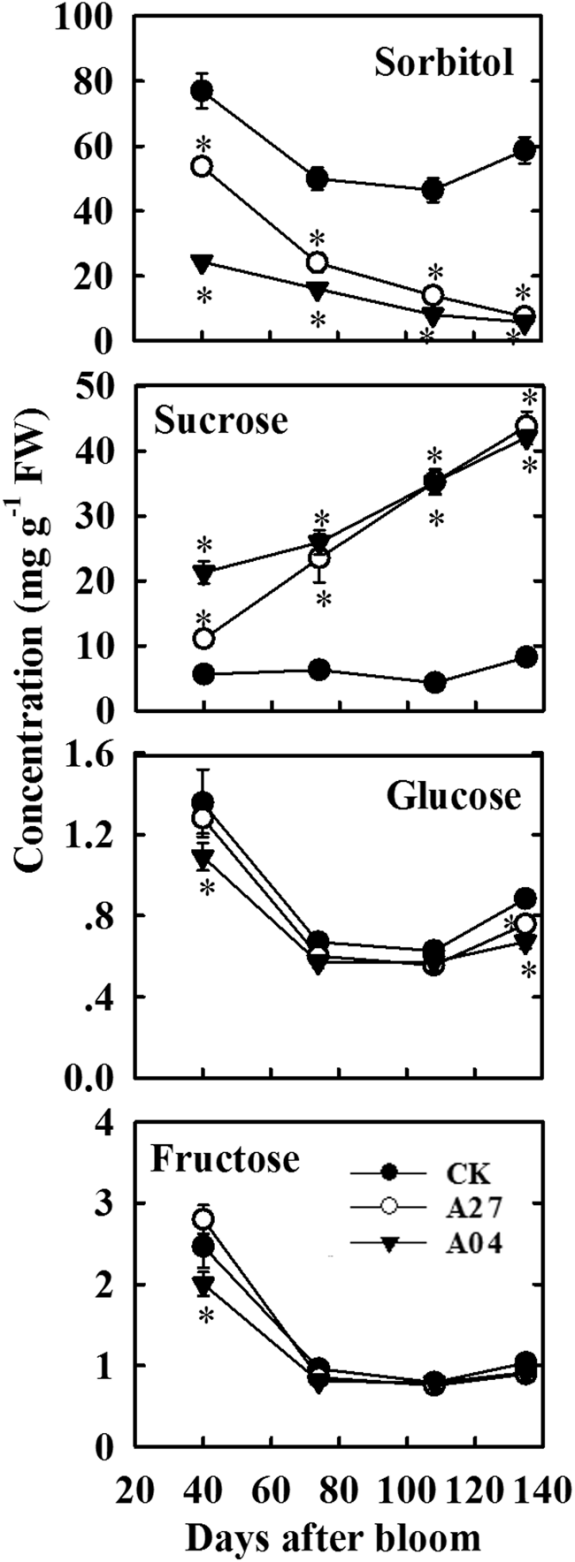
Concentrations of sorbitol, sucrose, fructose, and glucose in the leaves of the untransformed control (CK) and transgenic lines (A27 and A04) of “Greensleeves” apple during fruit development. Values are means of five replicates ± SD. An asterisk indicates a significant difference between transgenic lines (A27 or A04) and CK at *P* < 0.05 by Tukey’s test after analysis of variance

Antisense suppression of *A6PR* significantly decreased sorbitol concentration but increased sucrose concentration while largely maintaining fructose and glucose concentrations in source leaves throughout fruit development in the two transgenic lines (A27 and A04) relative to the untransformed CK (Fig. 1). Sorbitol concentration in the source leaves of antisense line A27 was decreased to ~70% initially and 13% at harvest of that detected in CK. For antisense line A04, sorbitol concentration was decreased to 32% initially and 10% at harvest of the CK level. By contrast, sucrose concentration in the source leaves of A27 and A04 was much higher than in CK throughout fruit development, with larger differences detected at later developmental stages (Fig. 1).

Concentrations of sorbitol and sucrose were also measured for source leaves, leaf petioles, and fruit pedicels at 75 days after bloom (DAB) (Table 1). Compared with CK, antisense lines A27 and A04 had lower concentration of sorbitol, higher concentration of sucrose, and lower molar ratio of sorbitol to sucrose in the source leaves, leaf petioles, and fruit pedicels. The abundance of sorbitol followed the order of source leaves > leaf petioles > fruit pedicels (Table 1).

**Table 1:** Concentrations (µmol g^−1^ FW) of sorbitol and sucrose and ratios of sorbitol to sucrose in leaves, leaf petioles, and fruit pedicels of the untransformed control (CK) and *A6PR* antisense transgenic lines (A27 and A04) of “Greensleeves” apple at 75 DAB

		Sorbitol	Sucrose	Sorbitol/sucrose
Leaves	CK	274.0 ± 19.24a	18.4 ± 2.75b	14.9 ± 0.75a
	A27	132.0 ± 15.96b	68.7 ± 10.78a	1.9 ± 0.26b
	A04	94.1 ± 4.02c	75.7 ± 5.48a	1.2 ± 0.18c
Leaf petioles	CK	98.9 ± 3.18a	3.3 ± 0.35c	30.0 ± 2.98a
	A27	69.8 ± 4.52b	12.4 ± 0.23b	5.6 ± 0.38b
	A04	58.8 ± 0.87c	15.2 ± 0.61a	3.9 ± 0.21c
Fruit pedicels	CK	78.6 ± 4.04a	6.6 ± 0.73b	11.9 ± 1.02a
	A27	50.8 ± 4.53b	12.1 ± 0.53a	4.2 ± 0.28b
	A04	47.2 ± 2.72b	13.4 ± 0.52a	3.5 ± 0.15c

Values are means of five replicates ± SD. Different letters within the same column indicate significant difference at *P* < 0.05 by Tukey’s test after analysis of variance

Net CO_2_ assimilation rates of bourse shoot leaves were monitored throughout the growing season and no significant difference was detected between the two antisense lines (A27 and A04) and the CK except for a significant drop in the antisense lines at fruit harvest (Fig. S1).

### Growth and respiration of the transgenic fruit

**Fig. 2:**
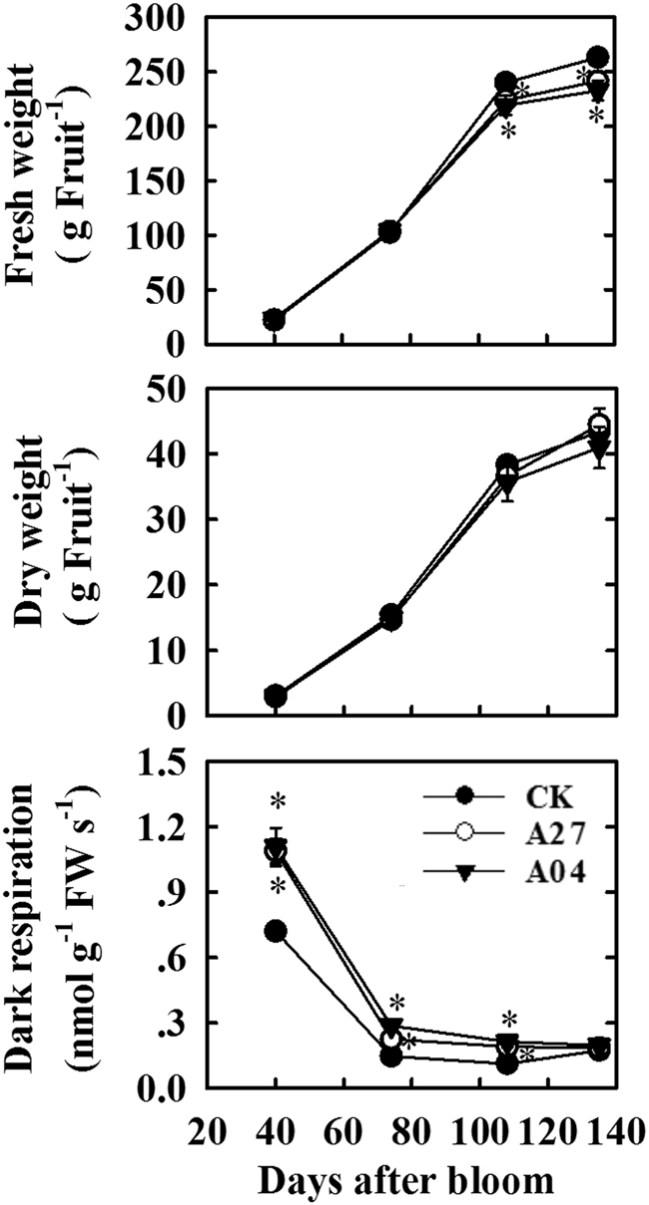
Fruit fresh weight, dry weight, and dark respiration of the untransformed control (CK) and transgenic lines (A27 and A04) of “Greensleeves” apple during fruit development. Values are means of five replicates ± SD. An asterisk indicates a significant difference between transgenic lines (A27 or A04) and CK at *P* < 0.05 by Tukey’s test after analysis of variance

Average fruit fresh weight did not differ significantly between the two antisense lines (A27 and A04) and CK during fruit development except for about a 10% lower value detected for A27 and A04 at 108 DAB and at harvest (134 DAB). Average fruit dry weight did not show any significant difference throughout fruit development (Fig. 2). Dark respiration was ~1.5–1.9-fold higher in A27 and A04 fruits than in CK fruits between 40 and 108 DAB during fruit development, but no significant difference was detected at harvest (Fig. 2).

Fruit yield per tree was significantly lower in the two antisense lines than in CK, largely due to lower average fruit weight at harvest as fruit number per tree was not significantly different between the two antisense lines and CK (Fig. S2).

### Concentrations of soluble sugars and starch in the transgenic fruit

**Fig. 3:**
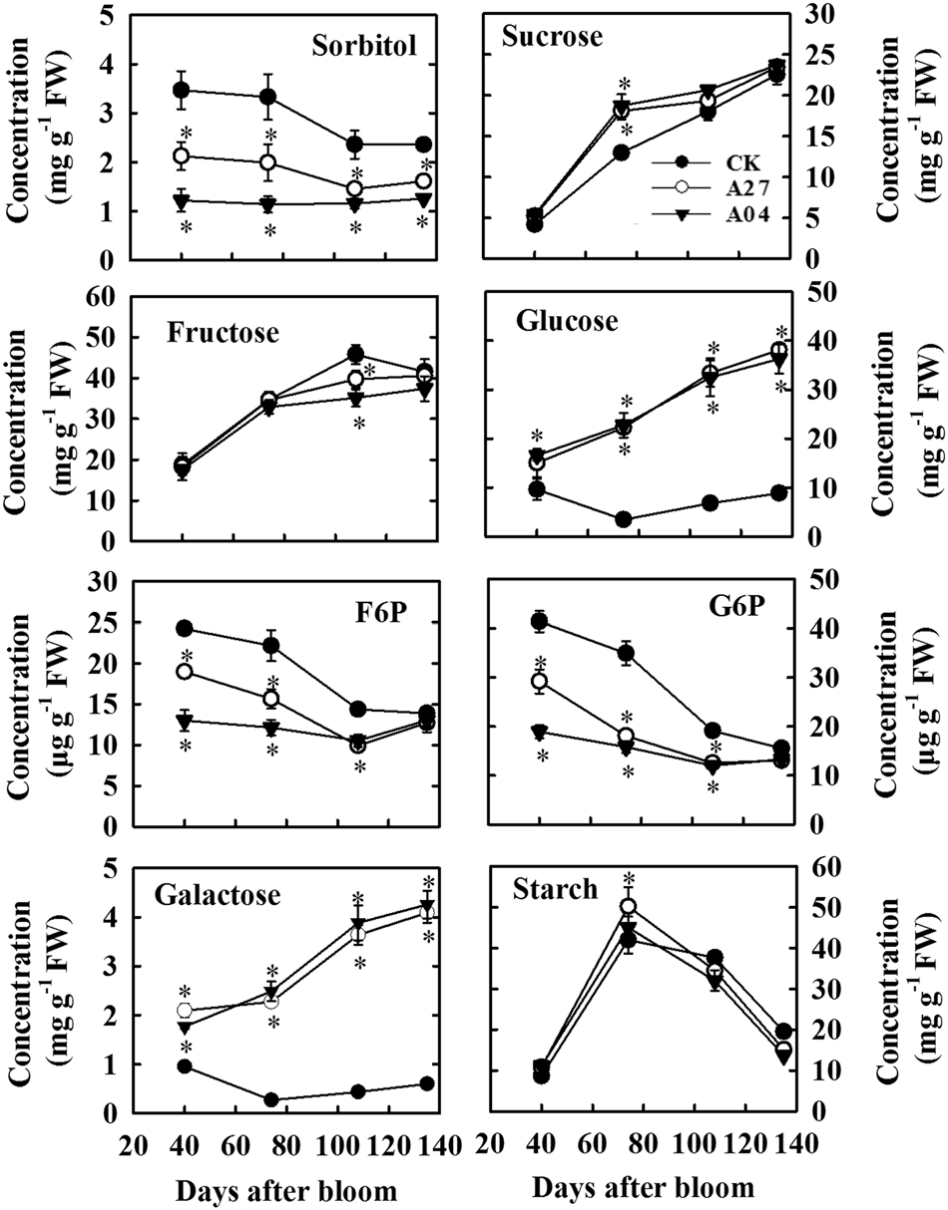
Concentrations of sorbitol, sucrose, fructose, glucose, fructose-6-phosphate (F6P), glucose-6-phosphate (G6P), galactose, and starch in the fruit of the untransformed control (CK) and transgenic lines (A27 and A04) of “Greensleeves” apple during fruit development. Values are means of five replicates ± SD. An asterisk indicates a significant difference between transgenic lines (A27 or A04) and CK at *P* < 0.05 by Tukey’s test after analysis of variance

Suppression of sorbitol synthesis in source leaves led to a significant decrease in sorbitol concentration in the fruit of two antisense lines A27 and A04 throughout fruit development, particularly in A04 (Fig. 3). However, sucrose concentration was similar in the fruits of the two antisense lines (A27 and A04) and CK during fruit development with a higher level detected in the transgenic fruit only at 74 DAB. Fructose concentration showed no difference between the transgenic fruit and CK except being slightly lower at 108 DAB in the transgenic fruit. Compared with CK, concentrations of glucose and galactose were much higher throughout fruit development, with larger differences detected at later developmental stages. Concentrations of G6P and fructose-6-phosphate (F6P) decreased during fruit development and were significantly lower in A27 and A04 than in CK from 40 to 108 DAB (Fig. 3). At fruit maturity (134 DAB), total soluble solids concentration was significantly higher in A27 (16.3%) and A04 (16.1%) than in CK (13.8%) (Fig. S2). Fruit starch concentration did not show obvious difference between the transgenic lines and CK before 74 DAB but was slightly lower in A27 and A04 than in CK after 108 DAB (Fig. 3)

### Activities of key enzymes in sugar metabolism in the transgenic fruit

**Fig. 4:**
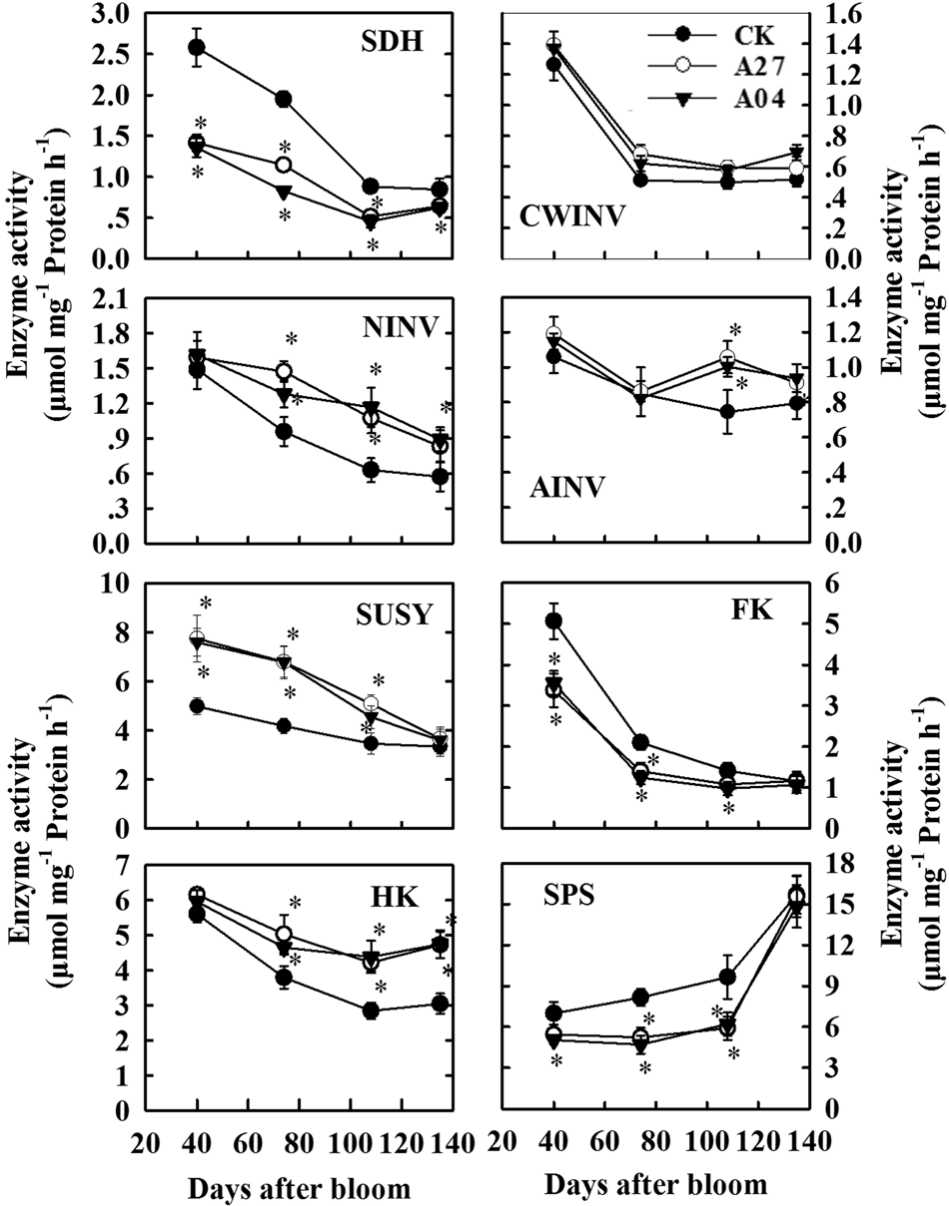
Activities of key enzymes involved in sugar metabolism in the fruit of the untransformed control (CK) and transgenic lines (A27 and A04) of “Greensleeves” apple during fruit development. SDH sorbitol dehydrogenase, CWINV cell wall invertase, NINV neutral invertase, vAINV vacuolar acid invertase, SUSY sucrose synthase, FK fructokinase, HK hexokinase, SPS sucrose phosphate synthase. Values are means of five replicates ± SD. An asterisk indicates a significant difference between transgenic lines (A27 or A04) and CK at *P* < 0.05 by Tukey’s test after analysis of variance

SDH activity decreased during fruit development and was significantly lower in both A27 and A04 than in CK at each developmental stage (Fig. 4). CWINV activity dropped dramatically from 40 to 74 DAB and then remained fairly constant to maturity, but no significant difference was detected between the two antisense lines and CK. NINV activity decreased throughout fruit development and was ~1.5–2.0-fold higher in A27 and A04 than in CK from 74 DAB to fruit maturity (Fig. 4). Vacuolar acid invertase (vAINV) activity showed no significant difference between the two antisense lines and CK except a slightly higher activity detected in A27 and A04 than in CK at 108 DAB. SUSY activity declined during fruit development and was significantly higher in A27 and A04 than in CK from 40 to 108 DAB. FK activity decreased during fruit development and was significantly lower in A27 and A04 than in CK at 40 and 74 DAB. HK activity decreased during fruit development and was significantly higher in both A27 and A04 than in CK from 74 to 134 DAB. SPS activity increased slightly from 40 to 108 DAB and then dramatically to fruit maturity, with a significantly lower activity detected in both A27 and A04 from 40 to 108 DAB.

### Expression of genes involved in sugar metabolism in the transgenic fruit

**Fig. 5:**
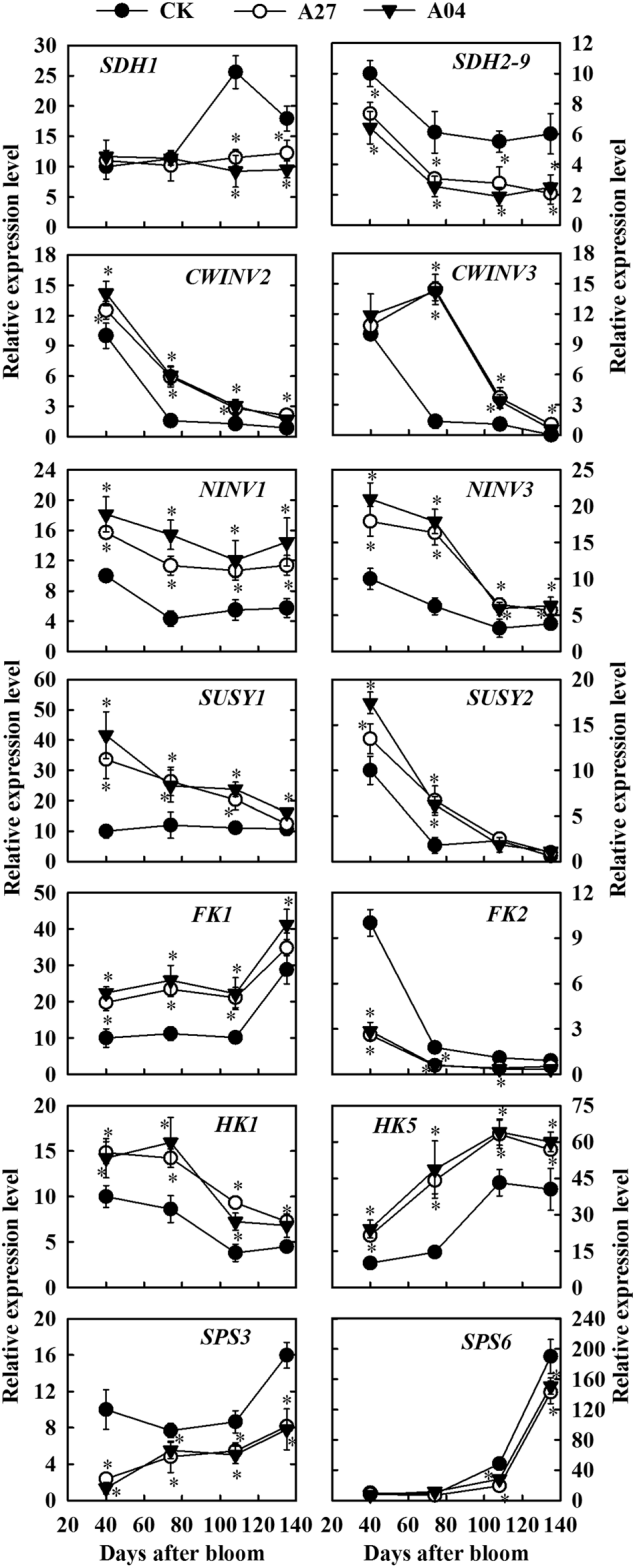
Relative mRNA expression levels of genes encoding key enzymes involved in sugar metabolism in the fruit of the untransformed control (CK) and transgenic lines (A27 and A04) of “Greensleeves” apple during fruit development. *MdSDH* *sorbitol dehydrogenase*, *MdCWINV* *cell wall invertase*, *MdNINV* *neutral invertase*, *MdSUSY* *sucrose synthase*, *MdFK* *fructokinase*, *MdHK* *hexokinase*, *MdSPS* *sucrose phosphate synthase*. Quantitative RT-PCR was performed with gene-specific primers except that a pair of universal primers was designed from the conserved cDNA region of *MdSDH2* to *MdSDH9*. For each sample, transcript levels were normalized with those of *Actin*, and the relative expression level for each gene was obtained via the ddCT method. Expression in 40-DAB fruit was designated as “10”. Values are means of three replicates ± SD. An asterisk indicates a significant difference between transgenic lines (A27 or A04) and CK at *P* < 0.05 by Tukey’s test after analysis of variance

In response to reduced supply of sorbitol from source leaves, *MdSDH1* transcript level in the fruit of both antisense lines A27 and A04 was lower than in CK at 108 DAB and 134 DAB and those of *MdSDH2-9* were lower throughout fruit development (Fig. 5). Transcript levels of *MdCWINV2*, *MdCWINV3*, *vAINV1*, and *vAINV2* decreased dramatically from 40 to 74 DAB in CK fruit (Fig. 5; Fig. S3), but in response to increased supply of sucrose, *MdCWINV2* transcript level was slightly higher in the transgenic fruit than in CK throughout fruit development, and the drop of transcript levels of *MdCWINV3* and *MdvAINV2* at 74 DAB observed in CK fruit was delayed in the transgenic fruit. Transcript levels of both *MdNINV1* and *MdNINV3* were significantly higher in the transgenic fruit (Fig. 5), but no difference was detected in the transcript level of *MdNINV2* between the transgenic fruit and CK fruit during fruit development (Fig. S3). Transcript levels of *MdSUSY1-3* were higher in the transgenic fruit than in CK during fruit development, particularly at the early stages (Fig. 5; Fig. S3). *MdFK1* transcript level was higher but *MdFK2* transcript level was lower in both antisense lines during fruit development (Fig. 5). No difference was detected in transcript levels of *MdFK3* or *MdFK4* between the two antisense lines and CK (Fig. S3). Transcript levels of six *MdHKs* were all upregulated in the transgenic fruit over CK either throughout fruit development (*MdHK1*, *MdHK5*, and *MdHK6*) or primarily at 74 DAB (*MdHK2-4*) (Fig. 5; Fig. S3). Transcript levels of *MdSPS3* and *MdSPS6* were lower in the transgenic fruit than in CK during fruit development (Fig. 5) whereas no difference was detected for the transcript level of *MdSPS1*, *MdSPS2*, *MdSPS4*, or *MdSPS5* (Fig. S3).

### Expression of genes involved in sugar transport in the transgenic fruit

**Fig. 6:**
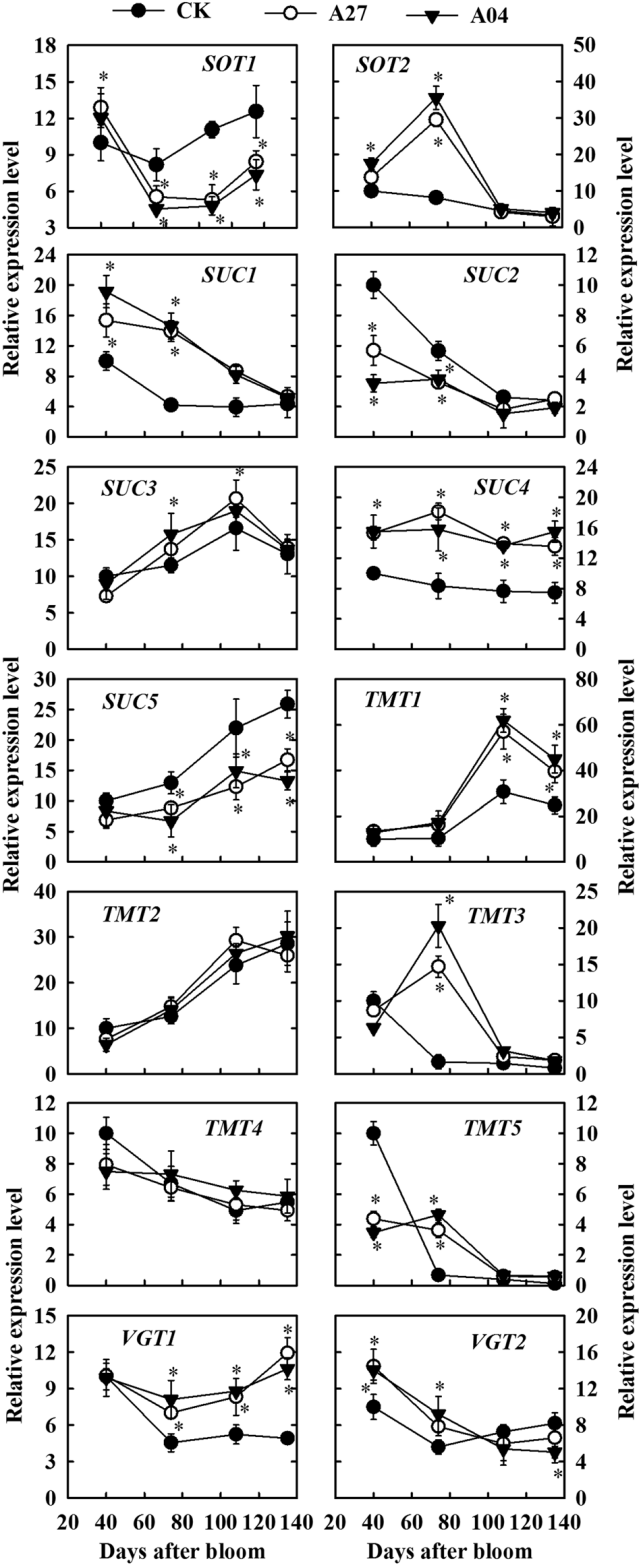
Relative mRNA expression levels of genes encoding sugar transporters in the fruit of the untransformed control (CK) and transgenic lines (A27 and A04) of “Greensleeves” apple during fruit development. *MdSOT* *sorbitol transporter*, *MdSUC* *sucrose carrier/transporter*, *MdTMT* *tonoplast monosaccharide transporter*, *MdvGT* *vacuolar glucose transporter*. Quantitative RT-PCR was performed with gene-specific primers. For each sample, transcript levels were normalized with those of *Actin*, and the relative expression level for each gene was obtained via the ddCT method. Expression in 40-DAB fruit was designated as “10”. Values are means of three replicates ± SD. An asterisk indicates a significant difference between transgenic lines (A27 or A04) and CK at *P* < 0.05 by Tukey’s test after analysis of variance

Compared with CK, the fruit of both antisense lines A27 and A04 had a lower transcript level of *sorbitol transporter 1* (*MdSOT1*) from 74 to 134 DAB, but a higher transcript level of *MdSOT2* at 74 DAB (Fig. 6). During fruit development, transcript levels of *sucrose transporter 1* (*MdSUC1*) and *MdSUC4* were higher in the transgenic fruit, whereas those of *MdSUC2* and *MdSUC5* were lower, with no difference detected in the transcript level of *MdSUC3* between the transgenic fruit and CK. Transcript level of *tonoplast monosaccharide transporter 1* (*MdTMT1*) was higher in the transgenic fruit at 108 DAB and 138 DAB and transcript levels of *MdTMT3* and *MdTMT5* were higher at 74 DAB, but no difference was detected in the transcript level of either *MdTMT2* or *MdTMT4* between the transgenic fruit and CK fruit during fruit development. Transcript level of *vacuolar glucose transporter 1* (*MdvGT1*) was higher from 74 DAB to harvest while that of *MdvGT2* was only slightly higher at 40 and 74 DAB (Fig. 6).

## Discussion

### Decreased sorbitol synthesis in the source leaves leads to a lower sorbitol but higher sucrose supply to fruit in the transgenic plants

Our data clearly showed that sorbitol concentration was significantly lower, whereas sucrose concentration was significantly higher in the source leaves of 5-year-old transgenic “Greensleeves” apple trees with antisense suppression of *A6PR* compared with the untransformed CK throughout fruit development. These results are consistent with those reported for the 1-year-old transgenic trees^13^. The higher sucrose concentration in the source leaves is an indication that a larger proportion of the photosynthetically fixed carbon ends up in sucrose over a 24-h period because most of the starch accumulated during the day breaks down for sucrose synthesis at night in the transgenic plants although no difference in the carbon flux to sucrose during the day was detected^13^. As both sorbitol and sucrose diffuse into SE-CC complex from mesophyll cells via plasmodesmata^16,17^, accumulation of a higher level of sucrose in leaves is expected to facilitate the transport of sucrose in the phloem when less sorbitol is translocated in the transgenic plants. The lower concentration of sorbitol and higher concentration of sucrose in both leaf petiole and fruit pedicel and a smaller ratio of sorbitol to sucrose (Table 1) indicate that significantly less sorbitol but much more sucrose is translocated from leaves to fruit in the transgenic trees, which is consistent with a lower sorbitol but a higher sucrose concentration in the phloem exudates collected from fruit pedicels of these plants. The total amount of carbon translocated to fruit is expected to be very similar between the transgenic lines and the CK because all the trees had a very similar cropload and no significant difference was detected in average fruit dry weight between the transgenic lines and the CK at fruit maturity (Fig. 2). These data clearly demonstrate that, when sorbitol synthesis is decreased in the source leaves, more sucrose is synthesized in the leaves and translocated to the fruit, thereby largely maintaining fruit growth and development. This is also consistent with the homeostasis of vegetative growth observed in the transgenic lines^13,23^.

The transgenic trees with decreased sorbitol synthesis grown under our experimental conditions were only slightly smaller after 5 years of growth than those of the untransformed CK (data not shown). This is consistent with comparable photosynthetic rates measured in the transgenic lines and the untransformed CK throughout the growing season (Fig. S1), with the lower rates detected only at fruit harvest being largely related to the leaf brown spots caused by *Alternaria alternata* in the transgenic lines^22^. However, Teo et al.^24^ found that the transgenic trees were much smaller than the CK trees. This discrepancy is likely due to differences in growing conditions between the two locations. As sorbitol is implicated in drought-stress tolerance in apple^25^, these trees might have experienced more drought stress under warm and dry conditions in California than under cool and humid conditions in upstate New York. In addition, as we strictly controlled cropload to a similar level each year by thinning flowers and young fruits, dry matter accumulation was not drastically different between the transgenic lines and the CK in our experiment.

### Both the Sucrose cycle and the sugar transport system in the transgenic fruit respond to a lower sorbitol but higher sucrose supply

In response to a decreased sorbitol supply from source leaves, both the transcript level and the activity of SDH decreased in the transgenic fruit, which is consistent with previous findings in apple fruit cortex tissues fed with sorbitol^26^ and in shoot tips and fruit of the transgenic trees^23,24^. As most of the fructose in apple fruit is converted from sorbitol by SDH, a significantly lower fructose level had been predicted in the transgenic fruit based on dramatically reduced import of sorbitol into the transgenic fruit and the associated lower SDH activity. However, the fructose level in the transgenic fruit was remarkably similar to that in the untransformed CK: no difference before 74 DAB and at harvest with only a slight difference detected at rapid fruit expansion (108 DAB) between the transgenic fruit and the CK (Fig. 3). This near homeostasis of fructose level in the transgenic fruit has clearly resulted from the response of the Sucrose cycle and the associated sugar transport system to increased availability of sucrose in the transgenic fruit, specifically, (1) more sucrose is taken up into parenchyma cells in fruit after phloem unloading; (2) more fructose is generated from sucrose breakdown by NINV and sucrose synthase and less fructose is phosphorylated by FK in the cytosol; and (3) tonoplast sugar transporters are upregulated to take up more hexoses into the vacuole.

In apple fruit, sucrose as well as sorbitol enters the parenchyma cells via the apoplastic pathway after being released from SE-CC complex^18^. In many species that employ apoplastic unloading for sucrose in sink cells, sucrose is mainly converted to glucose and fructose by CWINV in the cell wall space and then transported into the parenchyma cells by hexose transporters^9,27,28^. CWINV is typically considered as a sink-specific enzyme and its activity is usually very low in source leaves^27,29^. However, we found that, except for *MdCWINV3* in 40-DAB fruit, the expression of *MdCWINV*s was much lower in the fruit than in the shoot tips where sucrose unloading is symplastic^3^. In yeast cells expressing apple SOTs, sorbitol uptake is competitively inhibited by glucose and fructose but not by sucrose^30,31^. So we postulate that most sucrose is directly transported into the parenchyma cells by plasma membrane-bound SUCs in apple fruit to avoid inhibition of sorbitol uptake by sucrose-derived glucose and fructose. Increased sucrose import into transgenic fruit did not alter the activity of CWINV (Figs. 4 and 5) but significantly elevated the transcript levels of both *MdSUC1* (Group 4/SUT4 of the SUC family, high affinity sucrose transporter^32^) and *MdSUC4* (Group 3/SUT2 clade, low affinity transporter^3^), indicating that more sucrose is taken up into the parenchyma cells in the transgenic fruit.

There are two pathways for sucrose breakdown in the cytosol of fruit parenchyma cells: conversion to fructose and glucose by NINV or to fructose and UDP-glucose by SUSY^9^. The upregulation of transcript levels of *MdNINV1*, *MdNINV3*, and *MdSUSY1-3* and activities of NINV and SUSY in the transgenic fruit (Figs. 4 and 5), which is indicative of higher availability of sucrose in the cytosol, generates more fructose. This, combined with a lower *FK2* transcript level and a lower FK activity (Figs. 4 and 5), makes enough fructose available in the cytosol for accumulation in the vacuole of the transgenic fruit to largely compensate for the reduced level of sorbitol-derived fructose. The higher NINV activity is also expected to elevate the glucose level in the cytosol, which may have led to higher transcript levels of *MdHK*s and a higher HK activity through glucose signaling^33,34^ and a higher dark respiration rate in the transgenic fruit (Fig. 2). The higher HK activity detected in the transgenic fruit is similar to that of rice leaves in response to glucose manipulation^35^. However, we found that increases in both HK activity and the glucose concentration did not enhance, but rather diminished, the accumulation of G6P in the transgenic fruit. This is likely due to a decrease in F6P flux from phosphorylation of fructose along with an increase in dark respiration such that more G6P was reversibly converted to F6P. Our result is consistent with the finding that glucose derived from sucrose contributes to the hexose phosphate pool more than fructose derived from sorbitol or sucrose in the apple fruit^36^. Despite a higher glucose flux going through dark respiration in the transgenic fruit, more glucose is still available for transport into vacuole for accumulation as indicated by the 3–6-fold increase in glucose concentration in the transgenic fruit at harvest. Higher fruit glucose levels have also been reported for these antisense plants by Teo et al.^24^ but to a lesser degree. It is interesting that greater import of sucrose did not significantly increase its concentration in transgenic fruit except at 74 DAB. We think that two factors may have contributed to this near homeostasis of sucrose in the transgenic fruit. First, upregulation of sucrose breakdown described above uses more sucrose. Second, downregulation of *MdSPS3* and *MdSPS6* transcript levels and SPS activity in the transgenic fruit makes less sucrose re-synthesized from F6P and UDP-glucose.

While upregulation of SUSY in response to increased sucrose supply was observed in both fruit and shoot tips of the transgenic plants, NINV responded in the transgenic fruit but not in the shoot tips^23^. The exact reason for this difference is not known, but differences in sucrose concentration and/or presence of different isoforms of NINV between fruit parenchyma cells and shoot tips might exist^3^. Our findings on activities of SUSY, NINV, FK, HK, and SPS are not in agreement with those reported by Teo et al.^24^. We believe that the discrepancy might be related to the difference in the way fruit samples were taken. In our study, it took about 2 min to cut and freeze fruit samples on site in the orchard, but in Teo et al.^24^ all harvested fruits were placed on ice before being transported to the laboratory and it was only after several quality indices were measured that the cortical tissues were frozen for further analysis. Because import of sorbitol and sucrose into fruit stops upon detachment from the tree, both enzyme activity and gene expression may be altered if they are not frozen in liquid nitrogen in a very short period of time. In addition, strict cropload CK in our study as reflected in much larger fruit (average fresh fruit weight of 233 to 263 g in our study vs. around 160 g in Teo et al.^24^) might have made the difference between the transgenic fruit and the CK easier to be detected.

Most of the hexoses and sucrose in fruit parenchyma cells are stored in the central vacuole that occupies >80% of the cell volume^1^. The uptake of these sugars into the vacuole is carried out by sugar transporters located on the tonoplast. Transcript levels of *MdvGT1* and *MdvGT2*, both of which are vacuolar glucose transporters encoded by two *Malus* orthologs of *AtvGT*^37^, were higher in the transgenic fruit than in the CK (Fig. 6), suggesting that more glucose is transported into the vacuole of the transgenic fruit. This is consistent with the glucose concentration measured on bulk fruit samples (Fig. 3). In addition, tonoplast monosaccharide transporters (TMTs) can transport both glucose and fructose into the vacuoles, and *Arabidopsis* TMT1 activity for fructose is approximately 30% of that for glucose^38^. In the five *Malus* orthologs of *TMT*, it is possible that proteins encoded by *MdTMT1* and/or *MdTMT2* have high ability to transport fructose^3,19^, and the enhanced expression by *MdTMT1* in the transgenic fruit might indicate a regulatory response to the reduced flux of fructose derived from sorbitol. Alternatively, as fructose-specific TMTs have not been identified in fructose-accumulating fleshy fruits, the upregulation of *MdTMT1* could be triggered by higher levels of glucose derived from sucrose in the transgenic fruit. In addition to hexoses, the vacuoles in ripening apple fruit accumulate a high concentration of sucrose^1^. So far, no *SUC* has been identified to have proton-coupled antiport activity for loading sucrose into the vacuole^39^, but AtTMT1/2 probably represents a proton-coupled antiporter capable of transporting both glucose and sucrose into the vacuole^40^. A recent report on TMTs in sugar beet indicates that one of the two TMT2 proteins has developed specific affinity to sucrose and is responsible for sucrose accumulation in the taproots^41^. The expression patterns of both *MdTMT1* and *MdTMT2* are in general agreement with that of sucrose accumulation in our apple fruit.

It has been demonstrated that interruption of carbohydrate import into fruit by girdling^42^ or adjustment of cropload^43^ did not alter the fructose level in apple fruit. Contrasting light exposure did not appear to affect peel fructose level either^44^. The data obtained from the transgenic fruit in this study provides further evidence for supporting the idea that the Sucrose cycle and the associated transport system operates to maintain the homeostasis of fructose in the apple fruit. From an evolutionary perspective, having fructose homeostasis in the apple fruit may help seed dispersal for this species because fructose is the sweetest among all the soluble sugars present in fleshy fruits.

In conclusion, when sorbitol synthesis is decreased by antisense suppression of *A6PR* in the source leaves of apple trees, less sorbitol but more sucrose is transported from the leaves to the fruit. In response to the lower sorbitol/higher sucrose supply, sorbitol metabolism is downregulated, whereas breakdown of sucrose is upregulated in the transgenic fruit to compensate for the decreased flux of fructose derived from sorbitol. This altered sugar metabolism, together with corresponding changes in the sugar transport system, leads to near homeostasis of fructose and sucrose and much higher levels of glucose and galactose in the transgenic fruit. This study clearly demonstrates the metabolic flexibility and the advantages of having two transport carbohydrates in sorbitol-synthesizing Rosaceae tree fruit species and the central role of the Sucrose cycle and the sugar transport system in determining sugar metabolism and accumulation in fleshy fruits.

## Materials and methods

### Plant materials

Five-year-old trees of the untransformed CK and transgenic lines (A27 and A04) of “Greensleeves” apple (*Malus domestica* Borkh.) with antisense suppression of *A6PR* expression were used. A6PR activity in mature leaves of A27 and A04 was decreased to about 30% and 15% of that of CK, respectively^13^. All trees were grafted onto M.26 rootstocks and grown outdoors at Ithaca, NY, USA, under natural conditions, in 55-L plastic pots containing a sand:MetroMix 360 medium (1:2, v-v) (Scotts, Marysville, OH, USA). There were five replicates for each genotype with three trees each arranged in a completely randomized design. The trees were trained as a spindle system at a density of 1.5 × 3.5 m^2^. They were moved into a screen house for the entire bloom period to prevent pollen escape and hand-pollinated using mixed pollen of crab apple and several commercial varieties. The cropload of these trees was adjusted by hand-thinning to four fruits per cm^2^ trunk cross-sectional area at 10-mm king fruit size. During the growing season, the trees were supplied twice weekly with 15 mM N using Plantex® NPK (20–10–20) with micronutrients (Plantex Corp., Ontario, Canada). Fungicides and pesticides were sprayed at regular intervals throughout the growing season. At 40 DAB (near the end of cell division), 74 DAB (early stage of cell expansion), 108 DAB (late stage of cell expansion), and 134 DAB (maturity), fruits were sampled from the south side of the tree canopy between 12 noon and 2:00 P.M. under full sun exposure. On each sampling date, five replicates per genotype with at least six fruits each from three trees were harvested. The fruits were immediately weighed, cut into small pieces after removing the core, and frozen in liquid nitrogen on-site. The entire process took approximately 2 min. To estimate the levels of transport carbohydrates, five replicates of leaf petioles and fruit pedicels with four each were covered with aluminum foil for 7 days prior to sampling on 75 DAB and were frozen in liquid nitrogen along with mature leaves. All samples were stored at −80 °C.

### Measurements of leaf photosynthesis and fruit dark respiration

Net CO_2_ assimilation rates of bourse shoot leaves were measured using a CIRAS-1 portable photosynthesis system (PP Systems, Amesbury, MA, USA) with a broad leaf chamber at 5 tree developmental stages from 30 DAB to fruit harvest. On each sampling date, two bourse shoot leaves per replicate were measured at mid-day under full sun exposure and ambient temperature and relative humidity conditions.

Fruit respiration was measured with the CIRAS-1 gas exchange system connected to a custom-made chamber, which accommodated the entire fruit for each sample. In the early afternoon (from 1:00 to 3:30 P.M.) on each of those four sampling dates, fruits under full sun exposure were detached from the trees and dark-adapted at 25 °C for 30 min before taking respiration measurements, and one fruit per replicate was measured.

### Measurements of soluble sugars and starch

As described previously^3,45^, soluble sugars and hexose phosphates were extracted in 75% methanol with ribitol added as an internal standard and then derivatized sequentially with methoxyamine hydrochloride and *N*-methyl-*N*-trimethylsilyl-trifluoroacetamide. After derivatization, the metabolites were analyzed with an Agilent 7890 A GC/5975C MS system (Agilent Technology, Palo Alto, CA, USA) on a DB-5MS capillary column (20 m × 0.18 mm × 0.18 µm) with a 5 m Duraguard column (Agilent Technology). The tissue residue after 75% methanol extraction for gas chromatography–mass spectrometry analysis was re-extracted three times with 80% (v/v) ethanol at 80 °C, and the pellet was retained for enzymatic determination of starch as glucose equivalents^3^.

### Assays of enzyme activities

The exact methods described by Li et al.^3^ were used to extract and assay activities of SDH, CWINV, NINV, vAINV, SUSY, FK, HK, and SPS in fruit samples in this study. Soluble proteins were measured using Coomassie blue and enzyme activities were expressed on a protein basis.

### mRNA expression analysis

Quantitative reverse transcription-polymerase chain reaction (qRT-PCR) was used to analyze the transcript levels of the genes encoding key enzymes involved in sugar metabolism, *sorbitol dehydrogenase 1 and 2–9* (*SDH1* and *SDH2–9*), *cell wall invertase 2 and 3* (*CWINV2* and *CWINV3*), *neutral invertase 1–3* (*NINV1–3*), *vacuolar acid invertase 1–2* (*vAINV1–2*), *sucrose synthase 1–5* (*SUSY1–5*), *fructokinase 1–4* (*FK1–4*), *hexokinase 1–6* (*HK1–6*), and *sucrose phosphate synthase 1–6* (*SPS1–6*), and those encoding sugar transporters involved in sugar accumulation, *sorbitol transporters 1–2* (*SOT1–2*), *sucrose carriers/transporters 1–5* (*SUC1–5*), *tonoplast monosaccharide transporter 1–5* (*TMT1–5*), and *vacuolar glucose transporter 1–2* (*vGT1–2*). All the primers and procedures were the same as described previously^3^. Briefly, qRT-PCR was performed with an iScript cDNA Synthesis Kit (Bio-Rad Laboratories, Hercules, CA, USA) according to the manufacturer’s instructions. Amplified products were quantified by an iQ5 Multicolor Real-Time PCR Detection System (Bio-Rad) using an iQ SYBR Green Supermix Kit (Bio-Rad). Transcripts of *Actin* (CN938023) served to standardize the cDNA from our test genes. Data were analyzed per the ddCT method using the iQ5 2.0 standard optical system software.

## Electronic supplementary material

Suppl. Fig 1

Suppl. Fig 2

Suppl. Fig 3

## References

[1] Yamaki S (1984). Isolation of vacuoles from immature apple fruit flesh and compartmentation of sugars, organic acids, phenolic compounds and amino acids. Plant Cell Physiol..

[2] Colaric M, Veberic R, Stampar F, Hudina M (2005). Evaluation of peach and nectarine fruit quality and correlations between sensory and chemical attributes. J. Sci. Food Agric..

[3] Li M, Feng F, Cheng L (2012). Expression patterns of genes involved in sugar metabolism and accumulation during apple fruit development. PLoS ONE.

[4] Nguyenquoc B, Foyer CH (2001). A role for ‘futile cycles’ involving invertase and sucrose synthase in sucrose metabolism of tomato fruit. J. Exp. Bot..

[5] Katz E (2011). Label-free shotgun proteomics and metabolite analysis reveal a significant metabolic shift during citrus fruit development. J. Exp. Bot..

[6] Nardozza S (2013). Metabolic analysis of kiwifruit (*Actinidia deliciosa*) berries from extreme genotypes reveals hallmarks for fruit starch metabolism. J. Exp. Bot..

[7] Desnoues E (2014). Profiling sugar metabolism during fruit development in a peach progeny with different fructose-to-glucose ratios. BMC Plant Biol..

[8] Li M (2016). Proteomic analysis reveals dynamic regulation of fruit development and sugar and acid accumulation in apple. J. Exp. Bot..

[9] Ruan YL (2014). Sucrose metabolism: gateway to diverse carbon use and sugar signaling. Ann. Rev. Plant Biol..

[10] Bieleski RL (1969). Accumulation and translocation of sorbitol in apple phloem. Aust. J. Biol. Sci..

[11] Bieleski RL, Redgwell RJ (1985). sorbitol versus sucrose as photosynthesis and translocation products in developing apricot leaves. Aust. J. Plant Physiol..

[12] Escobar-Gutierrez AJ, Gaudillere JP (2007). Carbon partitioning in source leaves of peach, a sorbitol synthesizing species, is modified by photosynthetic rate. Physiol. Plant.

[13] Cheng L (2005). Antisense inhibition of sorbitol synthesis leads to up-regulation of starch synthesis without altering CO_2_ assimilation in apple leaves. Planta.

[14] Negm FB, Loescher WH (1981). Characterization of aldose 6-phosphate reductase (alditol 6-phosphate: NADP 1-oxidoreductase) from apple leaves. Plant Physiol..

[15] Zhou R, Cheng L, Wayne R (2003). Purification and characterization of sorbitol-6-phosphate phosphatase from apple leaves. Plant Sci..

[16] Reidel EJ, Rennie E, Amiard V, Cheng L, Turgeon R (2009). Phloem loading strategies in three plant species that transport sugar alcohols. Plant Physiol..

[17] Fu Q, Cheng L, Guo Y, Turgeon R (2011). Phloem loading strategies and water relations in trees and herbaceous plants. Plant Physiol..

[18] Zhang LY (2004). Evidence for apoplasmic phloem unloading in developing apple fruit. Plant Physiol..

[19] Wei X, Liu F, Chen C, Ma F, Li M (2014). The *Malus domestic*a sugar transporter gene family: identifications based on genome and expression profiling related to the accumulation of fruit sugars. Front. Plant Sci..

[20] Wu T (2015). Suppressing sorbitol synthesis substantially alters the global expression profile of stress response genes in apple (*Malus domestica*) leaves. Plant Cell Physiol..

[21] Meng D (2018). Decreased sorbitol synthesis leads to abnormal stamen development and reduced pollen tube growth via a MYB transcription factor, MdMYB39L, in apple (*Malus domestica*). New Phytol..

[22] Meng, D. et al. Sorbitol modulates resistance to *Alternaria alternata* by regulating the expression of an *NLR* resistance gene in apple. *Plant Cell*10.1105/tpc.18.00231 (2018). 10.1105/tpc.18.00231 PMC6096587 29871985

[23] Zhou R, Cheng L, Dandekar AM (2006). Down-regulation of sorbitol dehydrogenase and up-regulation of sucrose synthase in shoot tips of transgenic apple trees with decreased sorbitol synthesis. J. Exp. Bot..

[24] Teo G (2006). Silencing leaf sorbitol synthesis alters long distance partitioning and apple fruit quality. PNAS.

[25] Wang Z, Stutte GW (1992). The role of carbohydrates in active osmotic adjustment in apple under water stress. J. Am. Soc. Hortic. Sci..

[26] Archbold DD (1999). Carbohydrate availability modifies sorbitol dehydrogenase activity of apple fruit. Physiol. Plant.

[27] Sturm A (1999). Invertases, primary structures, functions, and roles in plant development and sucrose partitioning. Plant Physiol..

[28] Zhang DP, Lu YM, Wang YZ, Duan CQ, Yan HY (2001). Acid invertase is predominantly localized to cell walls of both the practically symplasmically isolated sieve element/companion cell complex and parenchyma cells in developing apple fruits. Plant Cell Environ..

[29] Palmer WM, Ru L, Jin Y, Patrick JW, Ruan YL (2016). Tomato ovary-to-fruit transition is characterized by a spatial shift of mRNAs for cell wall invertase and its inhibitor with the encoded proteins localized to sieve elements. Mol. Plant.

[30] Gao ZF (2003). Cloning, expression, and characterization of sorbitol transporters from developing sour cherry fruit and leaf sink tissues. Plant Physiol..

[31] Watari J (2004). Identification of sorbitol transporters expressed in the phloem of apple source leaves. Plant Cell Physiol..

[32] Peng CC, Xu YH, Xi RC, Zhao XL (2011). Expression, subcellular localization and phytohormone stimulation of a functional sucrose transporter (MdSUT1) in apple fruit. Sci. Hortic..

[33] Karve AA (2008). Expression and evolutionary features of the hexokinase gene family in *Arabidopsis*. Planta.

[34] Sheen J (2014). Master regulators in plant glucose signaling networks. J. Plant Biol..

[35] Cho JI (2009). Role of the rice hexokinases OsHXK5 and OsHXK6 as glucose sensors. Plant Physiol..

[36] Berüter. J, Feusi MES, Rüedi P (1997). Sorbitol and sucrose partitioning in the growing apple fruit. J. Plant Physiol..

[37] Aluri S, Büttner M (2007). Identification and functional expression of the *Arabidopsis thaliana* vacuolar glucose transporter 1 and its role in seed germination and flowering. PNAS.

[38] Wormit A (2006). Molecular identification and physiological characterization of a novel monosaccharide transporter from Arabidopsis involved in vacuolar sugar transport. Plant Cell.

[39] Braun DM, Slewinski TL (2009). Genetic control of carbon partitioning in grasses: roles of sucrose transporters and tie-dyed loci in phloem loading. Plant Physiol..

[40] Schulz A (2012). Proton-driven sucrose symport and antiport is provided by the vacuolar transporters SUC4 and TMT1/2. Plant J..

[41] Jung B (2015). Identification of the transporter responsible for sucrose accumulation in sugar beet tap roots. Nat. Plants.

[42] Berüter J, Feusi MES (1997). The effect of grinding on carbohydrate partitioning in the growing apple fruit. J. Plant Physiol..

[43] Klages K (2001). Diurnal changes in non-structural carbohydrates in leaves, phloem exudate and fruit in ‘Braeburn’ apple. Funct. Plant Biol..

[44] Li P, Ma F, Cheng L (2013). Primary and secondary metabolism in the sun-exposed peel and the shaded peel of apple fruit. Physiol. Plant.

[45] Wang H, Ma F, Cheng L (2010). Metabolism of organic acids, nitrogen and amino acids in chlorotic leaves of ‘Honeycrisp’ apple (*Malus domestica* Borkh) with excessive accumulation of carbohydrates. Planta.

