# Supplementary figures and images for: Sugar metabolism and accumulation in the fruit of transgenic apple trees with decreased sorbitol synthesis
Source: Hortic Res. 2018 Dec 1;5:60. doi: 10.1038/s41438-018-0064-8 (PMC6269491; doi:10.1038/s41438-018-0064-8)

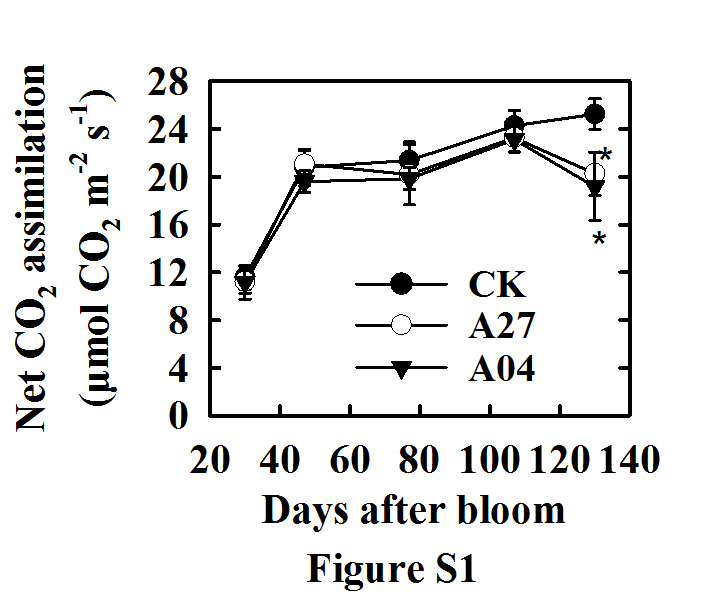

Supplement: Supplementary file 1 — Suppl. Fig 1 [file 41438_2018_64_MOESM1_ESM.jpg]

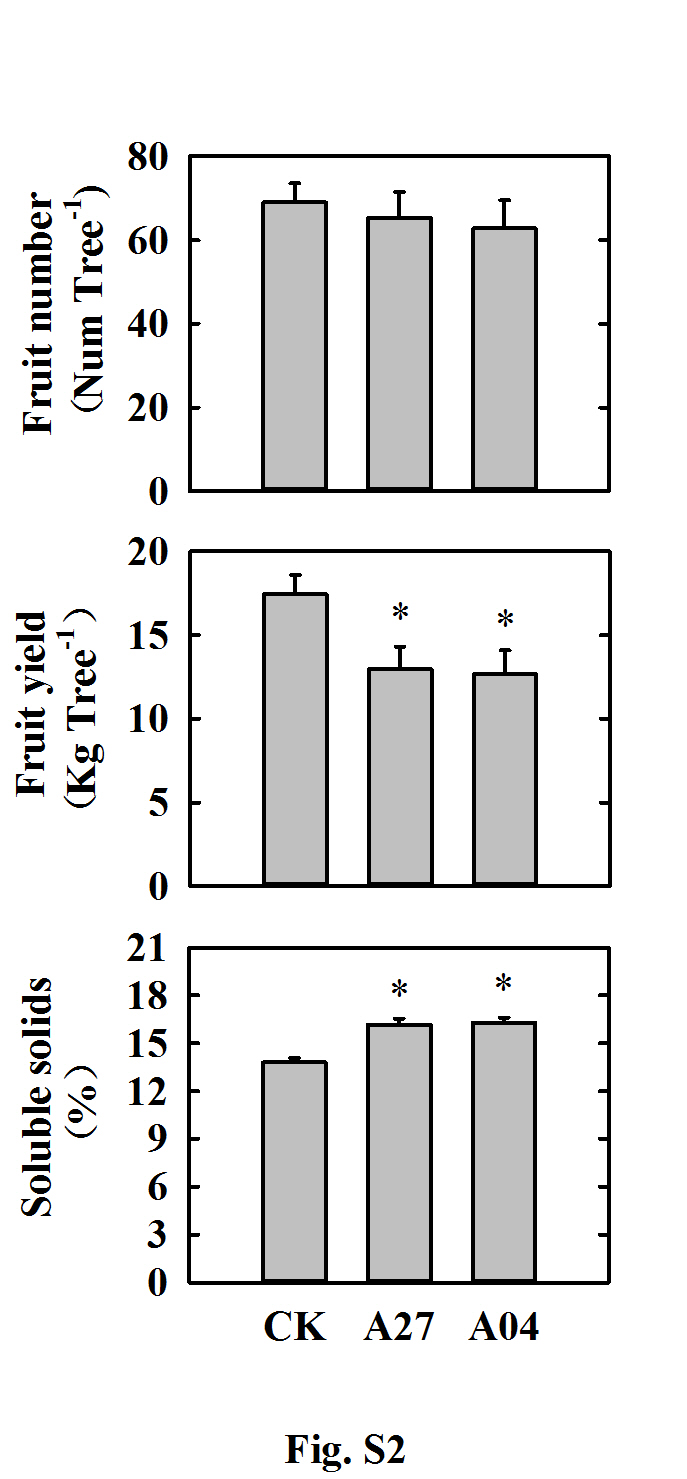

Supplement: Supplementary file 2 — Suppl. Fig 2 [file 41438_2018_64_MOESM2_ESM.jpg]

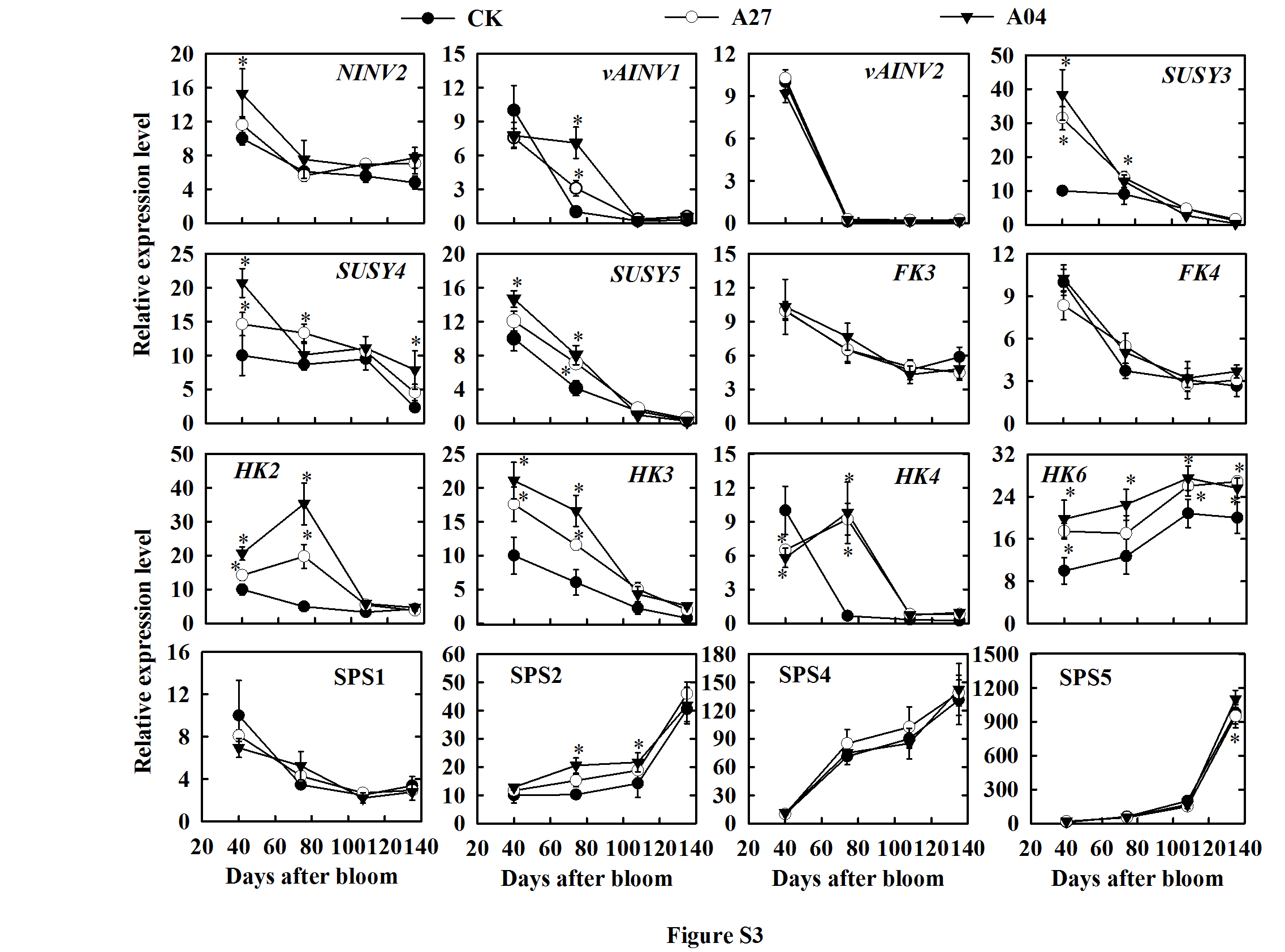

Supplement: Supplementary file 3 — Suppl. Fig 3 [file 41438_2018_64_MOESM3_ESM.jpg]
